# Etiology Exploration of Non-alcoholic Fatty Liver Disease From Traditional Chinese Medicine Constitution Perspective: A Cross-Sectional Study

**DOI:** 10.3389/fpubh.2021.635818

**Published:** 2021-05-12

**Authors:** Ke Zhu, Yongsong Guo, Chenghao Zhao, Shixin Kang, Jialiang Li, Jiexin Wang, Zhaohui Tang, Bing Lin, Weihong Li

**Affiliations:** ^1^Basic Medical College, Chengdu University of Traditional Chinese Medicine, Chengdu, China; ^2^Health Management Center, Affiliated Hospital of Chengdu University of Traditional Chinese Medicine, Chengdu, China

**Keywords:** phlegm-dampness constitution, non-alcoholic fatty liver disease, traditional Chinese medicine, constitutional theory, propensity score matching

## Abstract

**Background:** From the traditional Chinese medicine (TCM) constitution theory perspective, the phlegm-dampness constitution is thought to be closely related to the occurrence of non-alcoholic fatty liver disease (NAFLD). However, this viewpoint still lacks rigorous statistical evidence. This study aimed to test the association between the phlegm-dampness constitution and NAFLD.

**Methods:** We conducted a cross-sectional study. Participants were residents living in Chengdu, China, undergoing health checkups at the health management center of Affiliated Hospital of Chengdu University of Traditional Chinese Medicine between December 2018 and September 2020. TCM constitution type was diagnosed by DAOSH four examinations instrument, NAFLD was diagnosed according to the liver ultrasonography and medical history. Multivariate logistic regression and propensity score matching (PSM) were used to analyze a total of 1,677 qualified data.

**Results:** 1,037 participants had biased constitution(s), 67.8% of which had mixed constitutions (with at least two constitutions). Among 1,677 participants, the phlegm-dampness constitution was associated with the yang-deficiency, yin-deficiency, dampness-heat, qi-depression, and blood-stasis constitutions. The correlation coefficients were 0.11, 0.32, 0.42, 0.20, 0.14, respectively. Between the phlegm-dampness constitution and NAFLD, the odds ratio (OR) and the 95% confidence interval (CI) was 2.05 (1.57–2.69) in the crude model. After adjusting for age, gender, Body mass index (BMI), other biased constitutions, smoking, high blood pressure, diabetes, and dyslipidemia, the OR reduced to 1.51 (1.04–2.18). The associations of seven other biased TCM constitutions and NAFLD were not statistically significant in the fully adjusted model. The PSM analysis showed consistent results with the logistic regression.

**Conclusions:** Among eight biased TCM constitutions, the phlegm-dampness constitution is independently associated with NAFLD. We speculate the phlegm-dampness constitution is a risk factor of NAFLD. Longitudinal studies are needed to confirm this causal relationship in the future. In addition, inconsistent with some TCM practitioners' experience, we disagree that the blood-stasis constitution is associated with NAFLD.

## Introduction

Non-alcoholic fatty liver disease (NAFLD) is one of the most commonly encountered liver disorders worldwide (1), prevalence accounting for 25.24% (2). Its epidemic is believed to be driven by unhealthy lifestyles, aging, and genetics (3). The disease spectrum extends from liver steatosis to non-alcoholic steatohepatitis (NASH); the latter may progress to advanced liver fibrosis, cirrhosis, or hepatocellular carcinoma (HCC) (4). To date, NAFLD has become a significant public health concern. Research showed that NASH is the second leading cause of liver transplantation in the USA (5, 6), while NAFLD is classified as the most common risk factor for the development of HCC (7).

Traditional Chinese medicine (TCM) constitution theory has its unique understanding of NAFLD. A specific TCM constitution named phlegm-dampness constitution is thought to be an important risk factor for the occurrence of NAFLD (8). In China, the TCM constitution is applied to evaluate a person's health status. It refers to the integrative and relatively stable inherent qualities on morphological structures, physiological function, and psychological state, formed on the basis of natural endowment and acquired elements in the process of life, which is the personal characteristic of the human body adapting to natural and social environment formed in the process of human growth and development (9). The standard of Classification and Determination of TCM Constitution was established, officially released by the China Association of Chinese Medicine in 2009, and incorporated into The State's Basic Public Health Services Specification (2009 ed) promulgated by the Ministry of Health, The People's Republic of China (9, 10). There are nine TCM constitution types: one balanced constitution (gentleness) and eight biased constitutions (qi-deficiency, yang-deficiency, yin-deficiency, phlegm-dampness, dampness-heat, qi-depression, blood-stasis, and special diathesis constitutions).

People with the phlegm-dampness constitution have a series of comprehensive syndromes and signs, including phlegm-dampness coagulation in the body, obesity, greasy and soft lower abdomen, oily skin in the face, sticky and sweet taste in the mouth, and slippery pulse. They often have a low ability to adapt to the rainy season and humid environments (11). Some TCM practitioners pointed out that phlegm and dampness are the pathological basis of NAFLD by experience (12, 13). Epidemiological data also showed the phlegm-dampness constitution is one of the most common constitutions in NAFLD patients (14, 15). Additionally, experimental studies found phlegm-resolving and dampness-dispelling formula could alleviate the liver injury of NAFLD rats and NASH mice (16, 17). However, without setting the control group and adjusting confounding variables, these research studies are still insufficient to prove the correlation between the phlegm-dampness constitution and NAFLD. To clarify this correlation, we conducted this analytical cross-sectional study.

## Materials and Methods

### Study Sample

**Figure 1:**
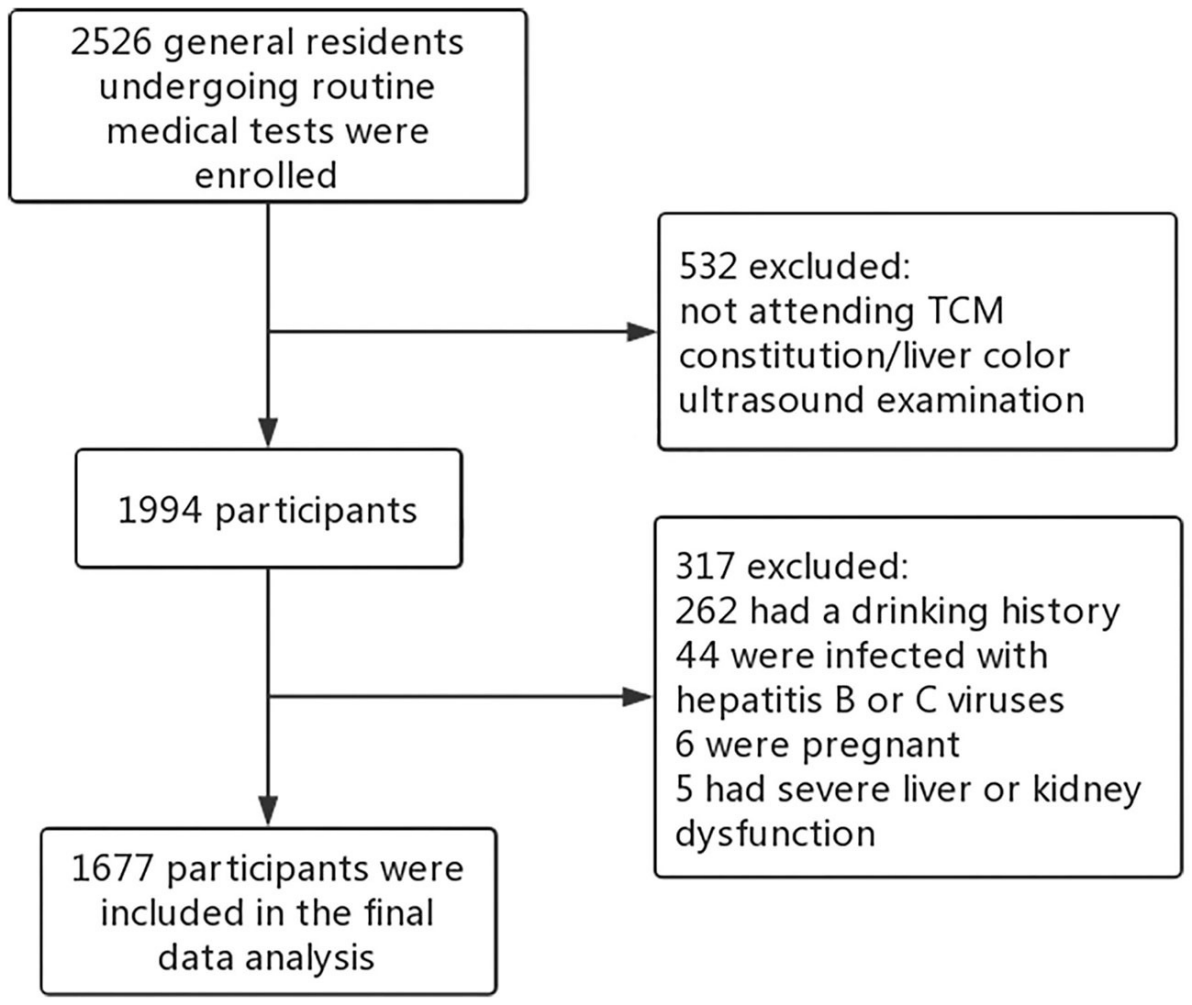
The flow chart of the study sample selection.

Participants included in this study were residents living in Chengdu, China, undergoing routine health checkups at the health management center of the Affiliated Hospital of Chengdu University of Traditional Chinese Medicine between December 2018 and September 2020. Most participants only attended one health checkup during the study period. For the few participants who attended health checkups more than once, we only collected their first health reports for data analysis. The checkup included anthropometric and basic clinical assessment (weight, height, blood pressure), biochemical (such as, blood routine examination, hepatic function, renal function, trace elements test), and imaging tests (such as, chest radiography, abdominal ultrasonography). Inclusion criteria: (1) aged over 18; (2) completing the TCM constitution questionnaire; (3) completing the color ultrasound examination of liver and gallbladder. Exclusion criteria: (1) history of alcohol consumption (drink more than once a week in the past year); (2) infected with hepatitis B or C virus; (3) severe liver and kidney dysfunction; (4) pregnant women. After screening, 1,677 participants' data were qualified for the final analysis (Figure 1). The ethics committee of the Affiliated Hospital of Chengdu University of Traditional Chinese Medicine approved the study (2018-KL050), and all participants provided written informed consent.

### Assessment of TCM Constitution

The participants' TCM constitutional types were diagnosed by DAOSH four examinations instrument (Shanghai Food & Drug Administration approval No. 20202200060 for medical devices). Participants filled in a questionnaire on this instrument, then the system automatically assessed their TCM constitutions according to the Nine Basic Constitutions Classification and Criteria in Chinese Medicine (18). The questionnaire includes 60 items, in which items of nine subscales (each subscale consists of 7–8 items) are interspersed irregularly. For each item, an appropriate answer was selected from a Likert scale (“no,” “occasionally,” “sometimes,” “often,” and “always,” scored 1–5). For each subscale, the original score was first calculated (original score = sum of the scores for each item) and then converted into the conversion score [(original score - number of subscale items)/(number of subscale items × 4) × 100]. The conversion score of each subscale ranged from 0–100 points. Gentleness constitution referred to the participants with conversion scores of the eight biased constitutions being <30 points, and the conversion score of the gentleness constitution being ≥60 points. A biased constitution was diagnosed if the conversion score of any constitution was ≥40 points (see the TCM constitution questionnaire in the Supplementary Material).

### Assessment of NAFLD

According to the definition of NAFLD, participants who met the following requirements were considered as NAFLD patients (19, 20): imaging evidence of hepatic steatosis; no excessive alcohol consumption (Alcohol consumption amounts to <30 g/d for men and <20 g/d for women); no specific diseases (such as, parenteral nutrition, viral hepatitis, drug hepatitis, and Wilson disease) that can lead to NAFLD. The diagnosis of fatty liver was assessed by experienced and trained radiologists who were blinded to the aim of the present study and the participants' clinical diagnosis. Ultrasonographic diagnosis of hepatic steatosis was determined by the presence of at least 2 of 3 abnormal findings on abdominal ultrasonography: diffusely increased echogenicity (“bright”) liver with liver echogenicity greater than kidney or spleen, vascular blurring, and deep attenuation of the ultrasound signal (21).

### Collection of Other Covariates

Other collected covariates included age, gender, height, weight, smoking history, previous medical history, blood pressure, blood glucose, and blood lipid. Experienced clinicians inquired about smoking history and previous medical history. Electronic instruments obtained standardized measurements of height, weight, and systolic and diastolic blood pressure. Body mass index (BMI) was calculated. Blood samples were collected from peripheral blood, and the laboratory department of the hospital detected blood glucose and blood lipid. A participant would be diagnosed as a diabetes patient if his/her concentration of fasting blood glucose ≥7.0 mmol/L or if he/she was currently taking any antidiabetic drugs. Hypertension was defined as the systolic blood pressure ≥140 mmHg or the diastolic blood pressure ≥90mmHg or who were currently undergoing antihypertensive treatments. Dyslipidemia was defined as an increase in plasma cholesterol (TC ≥ 6.2 mmol/L or LDL-C ≥ 4.1 mmol/L) or triglycerides (≥2.3 mmol/L), or a decrease in HDL cholesterol (≤1.0 mmol/L).

### Statistical Analysis

#### Sample Size Estimate

In this study, 14 independent variables were included in the multivariate logistic regression equation, containing gender, age, BMI, smoking status, hypertension, diabetes, dyslipidemia, phlegm-dampness, qi-deficiency, yang-deficiency, yin-deficiency, dampness-heat, qi-depression, blood-stasis, and special diathesis constitutions. According to the 10 events per variable (EPV) principle (22), the study required 140 NAFLD patients. About 29.81% of China's population was reported to have NAFLD (23), so that the estimated sample size would be 470. Apart from TCM constitutions, since we also need to analyze the participants' tongue manifestation and pulse condition in other studies. So we included participants as many as possible. This study eventually included 1,677 participants, and the sample size is big enough for logistic regression analysis.

#### Statistical Description and Inference

Continuous variables were expressed as mean ± standard deviation (normal distribution) or median (quartile) (skewed distribution), and categorical variables were expressed in frequency or as a percentage. The One-Way ANOVA (normal distribution), Kruskal-Wallis H (skewed distribution) test, and chi-square tests (categorical variables) were used to determine any statistical differences between the means and proportions of the groups. The polychoric correlation coefficient was calculated to determine the correlation between the phlegm-dampness constitution and other biased TCM constitutions. Logistic regression was used to evaluate the association between the phlegm-dampness constitution and NAFLD. According to the recommendation of the Strengthening the Reporting of Observational Studies in Epidemiology (STROBE) statement (24), we simultaneously showed the results of unadjusted, minimally adjusted analyses and those from fully adjusted analyses. In order to verify the stability of the results, we grouped participants according to whether they had the phlegm-dampness constitution, then used the PSM (propensity score matching) method to match the participants. Caliper was set to 0.01, with the proportion of 1:1, gender, age, BMI, smoking status, hypertension, diabetes, dyslipidemia, and other biased constitutions were matched. Subsequently, logistic regression was used to calculate the odds ratio and 95% confidence interval. All of the analyses were performed with the statistical software packages R (http://www.R-project.org, The R Foundation) and EmpowerStats (http://www.empowerstats.com,X&Y Solutions, Inc., Boston, MA). P-values <0.05 (two-sided) were considered statistically significant.

## Results

### Baseline Characteristics of Participants

**Figure 2:**
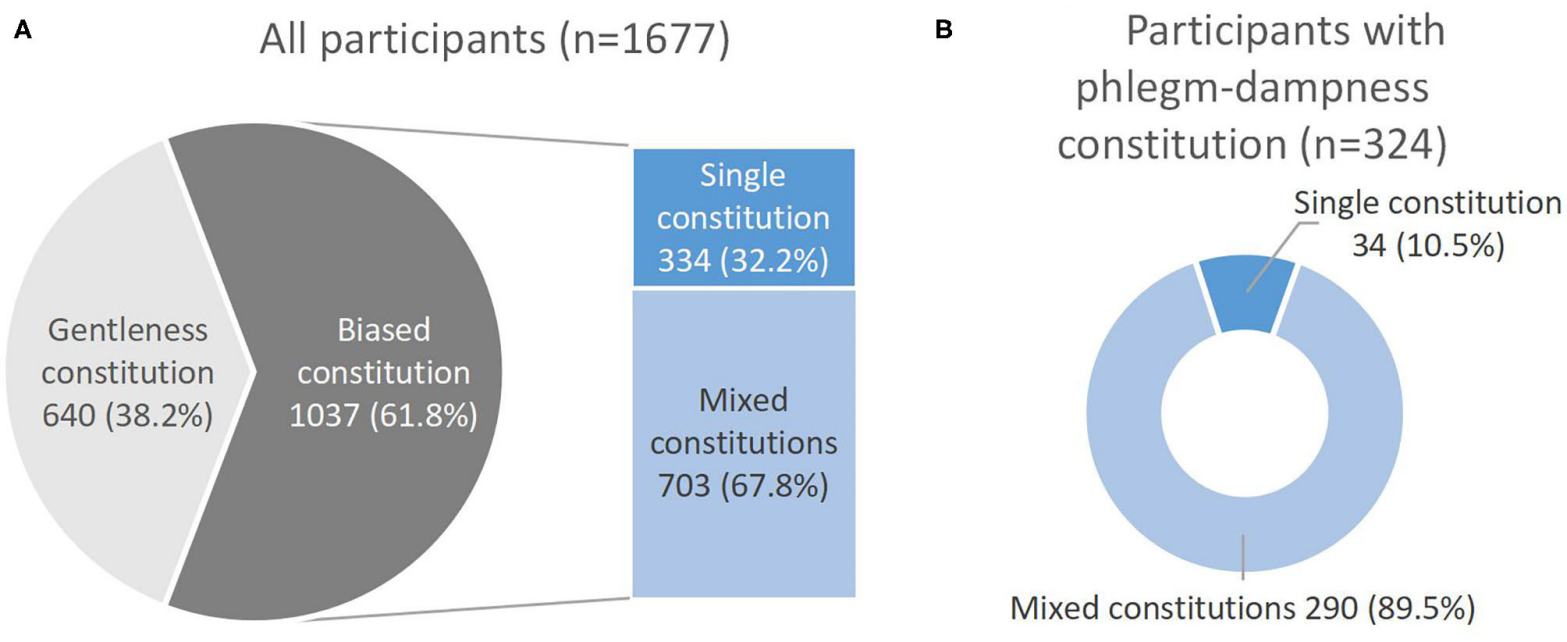
**(A)** Gentleness and biased constitution distribution in 1,677 participants and the proportion of single constitution and mixed constitutions in 1,037 participants with the biased constitution. **(B)** The proportion of single constitution and mixed constitutions in 324 participants with the phlegm-dampness constitution.

Among 1,677 participants, 365 (21.8%) were diagnosed with NAFLD. The average age of the participants was 48.3±10.4 years old, and about 57.4% of them are male. Compared with the non-NAFLD group, participants in the NAFLD group had a higher BMI, a higher proportion of male, phlegm-dampness, and dampness-heat constitutions, cigarette smoking, hypertension, diabetes and dyslipidemia, while a lower proportion of yang-deficiency, and blood-stasis constitutions (Table 1). In terms of TCM constitution distribution, the number of participants with the gentleness constitution was 640, accounting for 38.2%, while it with the biased constitution was 1,037, the proportion was 61.8%. Among participants with the biased constitution, 67.8% had mixed constitutions (with at least two constitutions). The majority of participants with the phlegm-dampness constitution had mixed constitutions, accounting for 89.5% (Figure 2).

**Table 1:** Baseline characteristics of 1,677 participants according to non-alcoholic fatty liver disease status.

**Characteristics**	**NAFLD**			
	**Total**	**No**	**Yes**	***P*-value**
Number	1,677	1,312	365	
Age (years, mean ± sd)	48.3 ± 10.4	48.4 ± 10.6	47.9 ± 9.6	0.43
Gender, male (*n*, %)	964 (57.4%)	676 (51.5%)	288 (78.9%)	<0.01
BMI (kg/m^2^, mean ± sd)	24.5 ± 3.4	23.7 ± 3.0	27.1 ± 3.0	<0.01
Cigarette smoking (*n*, %)	304 (19.2%)	204 (16.5%)	100 (29.2%)	<0.01
Hypertension (*n*, %)	205 (12.2%)	135 (10.3%)	70 (19.2%)	<0.01
Diabetes (*n*, %)	66 (3.9%)	34 (2.6%)	32 (8.8%)	<0.01
Dyslipidemia (*n*, %)	793 (47.3%)	616 (45.5%)	177 (54.6%)	<0.01
**TCM Constitutions (*****n*****, %)**				
Phlegm-dampness	324 (19.3%)	218 (16.6%)	106 (29.0%)	<0.01
Gentleness	640 (38.2%)	507 (38.6%)	133 (36.4%)	0.44
Qi-deficiency	323 (19.3%)	247 (18.8%)	76 (20.8%)	0.39
Yang-deficiency	387 (23.1%)	332 (25.3%)	55 (15.1%)	<0.01
Yin-deficiency	275 (16.4%)	215 (16.4%)	60 (16.4%)	0.98
Dampness-heat	364 (21.7%)	263 (20.0%)	101 (27.7%)	<0.01
Qi-depression	227 (13.5%)	182 (13.9%)	45 (12.3%)	0.45
Blood-stasis	173 (10.3%)	155 (11.8%)	18 (4.9%)	<0.01
Special diathesis	105 (6.3%)	82 (6.2%)	23 (6.3%)	0.97

*BMI, body mass index; NAFLD, non-alcoholic fatty liver disease*.

### Association Between the Phlegm-Dampness Constitution and Other Biased TCM Constitutions

Among 1,677 participants, chi-square and correlation analysis showed the phlegm-dampness constitution was positively associated with the yang-deficiency, yin-deficiency, dampness-heat, qi-depression, and blood-stasis constitution. The correlation coefficients were 0.11, 0.32, 0.42, 0.20, 0.14, respectively. In other words, participants with the phlegm-dampness constitution also tend to have the yang-deficiency, yin-deficiency, dampness-heat, qi-depression, or blood-stasis constitution (Table 2).

**Table 2:** Association between the phlegm-dampness constitution and seven other biased constitutions in 1,677 participants.

**Seven other biased constitutions (*n*, %)**	**Phlegm-dampness constitution**		**Correlation coefficient**	***P*-value**
	**No**	**Yes**		
Qi-deficiency	254 (18.8%)	69 (21.3%)	0.05	0.30
Yang-deficiency	297 (22.0%)	90 (27.8%)	0.11	0.03
Yin-deficiency	181 (13.4%)	94 (29.0%)	0.32	<0.01
Dampness-heat	228 (16.9%)	135 (41.7%)	0.42	<0.01
Qi-depression	161 (11.9%)	66 (20.4%)	0.20	<0.01
Blood-stasis	127 (9.4%)	46 (14.2%)	0.14	0.01
Special diathesis	88 (6.5%)	17 (5.2%)	−0.06	0.40

### Relationship Between the Phlegm-Dampness Constitution, Seven Other Biased Traditional Chinese Medicine Constitutions, and NAFLD

We applied multivariate logistic regression to analyze the association between TCM biased constitutions and NAFLD. TCM biased constitutions were set as the exposures, NAFLD was set as the outcome. For the phlegm-dampness constitution, without adjusting for any confounding variables, the crude model showed the odds ratio (OR) and the 95% confidence interval (CI) was 2.05 (1.57–2.69). After adjusting for gender and age, the OR and the CI was 1.94 (1.46–2.58). In the fully adjusted model, we adjusted gender, age, BMI, smoking status, hypertension, diabetes, dyslipidemia, qi-deficiency, yang-deficiency, yin-deficiency, dampness-heat, qi-depression, blood-stasis, and special diathesis constitutions. Although the OR reduced to 1.51 (1.04–2.18), there was still a statistical significance (*P* = 0.029). With the same analyzing process, the associations of seven other biased TCM constitutions and NAFLD were not statistically significant in the fully adjusted model (Table 3).

**Table 3:** Relationship between the phlegm-dampness constitution, seven other biased traditional Chinese medicine constitutions and non-alcoholic fatty liver disease in different models among 1,677 participants.

	**Crude model**		**Minimally adjusted model**		**Fully adjusted model**	
	**OR (95% CI)**	***P-*value**	**OR (95% CI)**	***P-*value**	**OR (95% CI)**	***P-*value**
**Phlegm-dampness**						
No	Reference		Reference		Reference	
Yes	2.05 (1.57, 2.69)	<0.001	1.94 (1.46, 2.58)	<0.001	1.51 (1.04, 2.18)	0.029
**Qi-deficiency**						
No	Reference		Reference		Reference	
Yes	1.13 (0.85, 1.51)	0.393	1.15 (0.85, 1.56)	0.379	1.32 (0.90, 1.94)	0.148
**Yang-deficiency**						
No	Reference		Reference		Reference	
Yes	0.52 (0.38, 0.72)	<0.001	0.58 (0.42, 0.81)	0.001	0.69 (0.46, 1.02)	0.064
**Yin-deficiency**						
No	Reference		Reference		Reference	
Yes	1.00 (0.73, 1.37)	0.981	1.12 (0.80, 1.56)	0.507	1.00 (0.66, 1.53)	0.982
**Dampness-heat**						
No	Reference		Reference		Reference	
Yes	1.53 (1.17, 2.00)	0.002	1.19 (0.89, 1.58)	0.244	0.93 (0.65, 1.35)	0.715
**Qi-depression**						
No	Reference		Reference		Reference	
Yes	0.87 (0.62, 1.24)	0.446	1.15 (0.79, 1.68)	0.465	1.00 (0.63, 1.60)	0.991
**Blood-stasis**						
No	Reference		Reference		Reference	
Yes	0.39 (0.23, 0.64)	<0.001	0.59 (0.35, 0.99)	0.048	0.58 (0.32, 1.05)	0.073
**Special diathesis**						
No	Reference		Reference		Reference	
Yes	1.01 (0.63, 1.63)	0.971	1.10 (0.66, 1.82)	0.725	1.34 (0.73, 2.45)	0.347

*Crude model adjusted for none. Minimally adjusted model adjusted for gender, age. Fully adjusted model adjusted for gender, age, BMI, smoking status, hypertension, diabetes, dyslipidemia, other biased constitutions. OR, odds ratio; CI, confidence interval*.

### Sensitivity Analysis

Besides, the PSM analysis method was applied to match the participants. 260 pairs of participants were matched successfully in the phlegm-dampness constitution group and the non-phlegm-dampness constitution group. There was no statistically significant difference between the paired phlegm-dampness group and the non-phlegm-dampness group in terms of age, gender, BMI, other biased TCM constitutions, smoking status, hypertension, diabetes, and dyslipidemia (Table 4). The prevalence of NAFLD was 36.5% in the phlegm-dampness constitution group, compared with 26.2% in the non-phlegm-dampness constitution group. After adjusting for confounding variables, the OR was 1.59 (1.02–2.50) (Table 5), which was consistent with the result of multivariate logistic regression analysis.

**Table 4:** Characteristics in non-phlegm-dampness and phlegm-dampness constitution groups after matching.

**Characteristics**	**Phlegm-dampness constitution**		***P-*value**
	**No**	**Yes**	
Number	260	260	
Age (years, mean ± sd)	49.34 ± 10.30	48.07 ± 11.12	0.18
Gender, male (*n*, %)	153 (58.8%)	164 (63.1%)	0.37
BMI	24.98 ± 3.52	25.32 ± 3.50	0.26
Cigarette smoking (*n*, %)	73 (28.1%)	81 (31.2%)	0.50
Hypertension (*n*, %)	43 (16.5%)	45 (17.3%)	0.91
Diabetes (*n*, %)	11 (4.2%)	16 (6.2%)	0.43
Dyslipidemia (*n*, %)	161 (61.9%)	164 (63.1%)	0.86
**TCM Constitutions (*****n*****, %)**			
Qi-deficiency	66 (25.4%)	59 (22.7%)	0.54
Yang-deficiency	85 (32.7%)	72 (27.7%)	0.25
Yin-deficiency	70 (26.9%)	65 (25%)	0.69
Dampness-heat	106 (40.8%)	110 (42.3%)	0.79
Qi-depression	47 (18.1%)	52 (20%)	0.66
Blood-stasis	39 (15%)	37 (14.2%)	0.90
Special diathesis	21 (8.1%)	15 (5.8%)	0.39

**Table 5:** Prevalence of non-alcoholic fatty liver disease in non-phlegm-dampness and phlegm-dampness constitution group after matching among 1,677 participants.

	**Phlegm-dampness constitution**		**OR of paired sample**	
	**No**	**Yes**	**Adjusted OR (95% CI)**	***P-*value**
NAFLD (*n*, %)	68 (26.2%)	95 (36.5%)	1.59 (1.02, 2.50)	0.042

*NAFLD, non-alcoholic fatty liver disease; OR, odds ratio; CI, confidence interval*.

## Discussion

### Main Findings

Our study results demonstrated the TCM phlegm-dampness constitution is indeed associated with NAFLD. People with the phlegm-dampness constitution have a 50% increase in the odds of having NAFLD than those without this constitution. After adjusting for potential confounding variables such as age, gender, BMI, lifestyle, other biased TCM constitutions, and previous history of chronic diseases by logistic regression, the odds ratio directions of both univariate and multivariate analysis were consistent. Subsequent sensitivity analysis also showed accordant results using the PSM method to match the participants, which further supported that the association of the phlegm-dampness constitution and NAFLD is stable.

Besides, we also found that people tend to have at least two TCM constitutions, namely mixed constitutions. People with the phlegm-dampness constitution often simultaneously have another TCM constitution, such as, the yang-deficiency, yin-deficiency, dampness-heat, qi-depression, or blood-stasis constitution. Because the coexisting biased constitution may also affect the diseases of the researchers' concern, therefore, when analyzing the relationship between a specific TCM constitution and a certain disease, other coexisting biased constitutions should be regarded as confounding variables for control. Otherwise, false positive or false negative results may be obtained. Among the published TCM constitution studies, the adjustment of other biased constitutions is often neglected. To promote the rigor and reliability of TCM constitution research, we hope that future researchers will pay attention to the confounding effect of other biased constitutions.

### Comparison With Other Studies

In line with prior descriptive cross-sectional studies (14, 15), we also found the qi-deficiency, phlegm-dampness, dampness-heat, and gentleness constitution are the common TCM constitutions in NAFLD patients. Based on personal clinical experience and these cross-sectional surveys, many TCM practitioners and researchers believe that the internal retention of phlegm and dampness are the etiology and pathogenesis of NAFLD (12, 13). Although, our study results could not yet demonstrate this empirical theory, we have proved the correlation between the phlegm and dampness constitution and NAFLD for the first time. It sets the basis for the further casual relationship investigation.

In addition to phlegm and dampness, blood stasis is also regarded as NAFLD's etiology and pathogenesis (12, 13), suggesting the blood-stasis constitution is associated with NAFLD. However, our data do not support this viewpoint. In our study, the association between the blood-stasis constitution and NAFLD neither has clinical significance or statistical significance. Since the theory released by TCM practitioners is a theoretical derivation and has not been tested before, according to our results, we think there is still no sufficient evidence to prove the blood-stasis constitution's causal effect on NAFLD.

### Potential Explanations

Based on the expert opinion that the biased TCM constitution is the basis of diseases (11), we speculate that the phlegm-dampness constitution is a status appearing before the occurrence of NAFLD. The correlation between the phlegm-dampness constitution and NAFLD might be due to some common pathological basis they have at the genetic level. It was found that the PPARGC1A knockout mice exhibited marked hepatic steatosis due to a combination of reduced mitochondrial respiratory capacity and increased expression of lipogenic genes (25). Clinical study also showed a significant association between genetic variations in PPARGC1A and NAFLD, which suggested PPARGC1A polymorphism and lower expression of PPARGC1A mRNA in the liver are an important genetic contribution to the etiology of NAFLD (26). In addition, ABCA1 and ACSL4 genes are reported to be associated with NAFLD as well (27–29). Coincidentally, some people with the phlegm-dampness constitution are also accompanied by the abnormal expression of above genes (30–32). Thus, we hypothesize that PPARGC1A, ABCA1, ACSL4 genes express abnormally in some patients due to either congenital defect or acquired unhealthy lifestyles, afterwards the patients will get into a phlegm-dampness constitution state. If this pathological state continues for long, NAFLD may eventually occur.

### Clinical Value

By revealing the correlation between the phlegm-dampness constitution and NAFLD, this study brings modern medicine community the TCM perspective on NAFLD, which could facilitate the communication between modern medicine and traditional medicine. It will also promote further longitudinal studies, which has certain guiding significance for the prevention and treatment of NAFLD. In terms of prevention, if future longitudinal studies prove that the phlegm-dampness constitution is a high-risk factor for the onset of NAFLD, intervention measures including some specific diet, exercise, drugs, and acupuncture will help prevent the disease by resolving phlegm and dispelling dampness. In terms of treatment, correcting patients' phlegm-dampness constituion through TCM therapy is expected to provide new ideas for modern medical treatment of NAFLD.

### Strengths and Limitations

This study's strengths are that it has a large sample size, and the data analysis process is rigorous. Two statistical analysis methods were adopted to control the confounding variables, and consistent results were obtained, indicating that the results are stable and reliable. Also, within all the clinical studies on TCM constitutions, we first noticed the confounding effect of coexisting biased constitutions and controlled it.

Meanwhile, our study has the following four limitations: (1) this is a single-center study, and no random sampling was conducted, so participants in this study may not represent all the general population of China; (2) because of the cross-sectional nature, participants' TCM constitution and NAFLD status were evaluated at the same time point, which could only prove the correlation between the phlegm-dampness constitution and NAFLD, rather than the causal conclusion (33); (3) the diagnosis of NAFLD was based on the ultrasound examination instead of the pathological biopsy, CT, or MR examination, some cases might be missed. This would lower the prevalence of NAFLD and affect the estimated OR value; (4) without randomized grouping, some unknown confounding variables were not collected and might be distributed unevenly between the two groups.

## Conclusions

Among eight biased TCM constitutions, the phlegm-dampness constitution is independently associated with NAFLD. Based on the TCM constitution theory, we speculate the phlegm-dampness constitution is a risk factor of NAFLD. Further longitudinal studies are needed to confirm this causal relationship. Additionally, inconsistent with some TCM practitioners' experience, we disagree that the blood-stasis constitution is associated with NAFLD. Besides, most people have mixed TCM constitutions, and the phlegm-dampness constitution is often accompanied by the yang-deficiency, yin-deficiency, dampness-heat, qi-depression, and blood-stasis constitution. Future clinical studies on the TCM constitution should notice the confounding effect of coexisting biased constitutions.

## Data Availability Statement

The original contributions presented in the study are included in the article/Supplementary Material, further inquiries can be directed to the corresponding author/s.

## Ethics Statement

The studies involving human participants were reviewed and approved by Affiliated Hospital of Chengdu University of Traditional Chinese Medicine. The patients/participants provided their written informed consent to participate in this study.

## Author Contributions

KZ and YG collected the clinical data and conducted statistical analysis. CZ, SK, JL, JW, and ZT participated in the data collection and the establishment of the database. KZ wrote the original draft. BL and WL were responsible for designing the study and reviewing the draft. All authors contributed to the article and approved the submitted version.

## Conflict of Interest

The authors declare that the research was conducted in the absence of any commercial or financial relationships that could be construed as a potential conflict of interest.

## References

[1] Sheka AC Adeyi O Thompson J Hameed B Crawford PA Ikramuddin S. Nonalcoholic steatohepatitis: a review. JAMA. (2020) 323:1175–83. 10.1001/jama.2020.2298 32207804

[2] Younossi ZM Koenig AB Abdelatif D Fazel Y Henry L Wymer M. Global epidemiology of nonalcoholic fatty liver disease-Meta-analytic assessment of prevalence, incidence, and outcomes. Hepatology. (2016) 64:73–84. 10.1002/hep.28431 26707365

[3] Stefan N Häring H Cusi K. Non-alcoholic fatty liver disease: causes, diagnosis, cardiometabolic consequences, and treatment strategies. Lancet Diabetes Endocrinol. (2019) 7:313–324. 10.1016/S2213-8587(18)30154-2 30174213

[4] Zhou J Zhou F Wang W Zhang XJ Ji YX Zhang P. Epidemiological features of NAFLD from 1999 to 2018 in China. Hepatology. (2020) 71:1851–64. 10.1002/hep.31150 32012320

[5] Noureddin M Vipani A Bresee C Todo T Kim IK Alkhouri N. NASH leading cause of liver transplant in women: updated analysis of indications for liver transplant and ethnic and gender variances. Am J Gastroenterol. (2018) 113:1649–59. 10.1038/s41395-018-0088-6 29880964 PMC9083888

[6] Stine JG Wentworth BJ Zimmet A Rinella ME Loomba R Caldwell SH. Systematic review with meta-analysis: risk of hepatocellular carcinoma in non-alcoholic steatohepatitis without cirrhosis compared to other liver diseases. Aliment Pharmacol Ther. (2018) 48:696–703. 10.1111/apt.14937 30136293 PMC7495494

[7] Younes R Bugianesi E. Should we undertake surveillance for HCC in patients with NAFLD? J Hepatol. (2018) 68:326–34. 10.1016/j.jhep.2017.10.006 29122695

[8] Sun J Sun M Yuan J Wu J Liu Q. Exploration on the distribution of phlegm -dam pness constitution of nonalcoholic fatty liver disease based on soil obstructed and wood obstructec. China J Tradit Chin Med Pharm. (2020) 35:956–8.

[9] Li LR Wang Q Wang J Wang QF Yang LL Zheng LY. Feasibility of assessing health state by detecting redox state of human body based on Chinese medicine constitution. Chin J Integr Med. (2016) 22:635–40. 10.1007/s11655-015-2327-7 26712210

[10] Wang J Li Y Wang Q. Identification of chinese medicine constitution in public health services. Chin J Integr Med. (2019) 25:550–3. 10.1007/s11655-016-2740-6 28028719

[11] Wang J Wang Q Li L Li Y Zhang H Zheng L. Phlegm-dampness constitution: genomics, susceptibility, adjustment and treatment with traditional Chinese medicine. Am J Chin Med. (2013) 41:253–62. 10.1142/S0192415X13500183 23548117

[12] Dong H Lu F Zhao L. Chinese herbal medicine in the treatment of nonalcoholic fatty liver disease. Chin J Integr Med. (2012) 18:152–60. 10.1007/s11655-012-0993-2 22311412

[13] Cai M Yang Y. Clinical experience in TCM differential treatment of fatty liver. J Tradit Chin Med. (2007) 27:115–6. 17710807

[14] Cai J Wang M. TCM constitution distribution characteristics of non-alcoholic simple fatty liver (in Chinese). World Latest Med Inf. (2017) 17:208–9. 10.19613/j.cnki.1671-3141.2017.102.109

[15] He Y Wang G Peng H. Analysis of TCM constitution distribution in 393 patients with nonalcoholic fatty liver disease (in Chinese). Jiangxi J Tradit Chin Med. (2019) 50:45–7.

[16] Zhao SP Wu ZS Chen Y Liang X Bao L Li P. Protective effect of Hua Tan Qu Shi decoction against liver injury in rats with nonalcoholic fatty liver disease. Biomed Pharmacother. (2017) 91:181–90. 10.1016/j.biopha.2017.04.099 28458156

[17] Leng J Huang F Hai Y Tian H Liu W Fang Y. Amelioration of non-alcoholic steatohepatitis by Qushi Huayu decoction is associated with inhibition of the intestinal mitogen-activated protein kinase pathway. Phytomedicine. (2020) 66:153135. 10.1016/j.phymed.2019.153135 31790895

[18] Wang Q. The Foundation of the Classification Diagnosis Standards for the Constitutions of TCM. China Standardization. (2009). p. 16–26.

[19] Marchesini G Day CP Dufour J-F Canbay A Nobili V Ratziu V. EASL-EASD-EASO clinical practice guidelines for the management of non-alcoholic fatty liver disease. J Hepatol. (2016) 64:1388–402. 10.1016/j.jhep.2015.11.004 27062661

[20] Wan Y Ma X Wang B Li Y Ren W Zhuang H. Guideline of prevention and treatment for nonalcoholic fatty liver disease: a 2018 update (in Chinese). J Clin Hepatol. (2018) 34: 947–57. 10.3760/cma.j.issn.1007-3418.2018.03.008

[21] Farrell GC Chitturi S Lau GK Sollano JD. Guidelines for the assessment and management of non-alcoholic fatty liver disease in the Asia-Pacific region: executive summary. J Gastroenterol Hepatol. (2007) 22:775–7. 10.1111/j.1440-1746.2007.05002.x 17565629

[22] Frank E Harrell J. Regression Modeling Strategies: With Applications to Linear Models, Logistic Regression, and Survival Analysis. New York, NY: Springer-Verlag (2001). 10.1007/978-1-4757-3462-1

[23] Li J Zou B Yeo YH Feng Y Xie X Lee DH. Prevalence, incidence, and outcome of non-alcoholic fatty liver disease in Asia, 1999–2019: a systematic review and meta-analysis. Lancet Gastroenterol Hepatol. (2019) 4:389–98. 10.1016/S2468-1253(19)30039-1 30902670

[24] von Elm E Altman DG Egger M Pocock SJ Gøtzsche PC Vandenbroucke JP. The strengthening the reporting of observational studies in epidemiology (STROBE) statement: guidelines for reporting observational studies. LANCET. (2007) 370:1453–7. 10.1016/S0140-6736(07)61602-X 18064739

[25] Leone TC Lehman JJ Finck BN Schaeffer PJ Wende AR Boudina S. PGC-1α deficiency causes multi-system energy metabolic derangements: muscle dysfunction, abnormal weight control and hepatic steatosis. PLoS Biol. (2005) 3:e101. 10.1371/journal.pbio.0030101 15760270 PMC1064854

[26] Yoneda M Hotta K Nozaki Y Endo H Uchiyama T Mawatari H. Association between PPARGC1A polymorphisms and the occurrence of nonalcoholic fatty liver disease (NAFLD). BMC Gastroenterol. (2008) 8:27. 10.1186/1471-230X-8-27 18588668 PMC2453128

[27] Wang C Liu S Lu L Liao S Yue H Dong Q. Association between four ABCA1 gene polymorphisms and risk of non-alcoholic fatty liver disease in a chinese han population. Hepat Mon. (2018) 18:e66149. 10.5812/hepatmon.66149

[28] Zhang RN Pan Q Zheng RD Mi YQ Shen F Zhou D. Genome-wide analysis of DNA methylation in human peripheral leukocytes identifies potential biomarkers of nonalcoholic fatty liver disease. Int J Mol Med. (2018) 42:443–52. 10.3892/ijmm.2018.3583 29568887

[29] Vega-Badillo J Gutierrez-Vidal R Hernandez-Perez HA Villamil-Ramirez H Leon-Mimila P Sanchez-Munoz F. Hepatic miR-33a/miR-144 and their target gene ABCA1 are associated with steatohepatitis in morbidly obese subjects. Liver Int. (2016) 36:1383–91. 10.1111/liv.13109 26945479

[30] Li L Feng J Yao H Xie L Chen Y Yang L. Gene expression signatures for phlegm-dampness constitution of Chinese medicine. Sci China Life Sci. (2017) 60:105–7. 10.1007/s11427-016-0212-9 27928700

[31] Wu Y Cun Y Dong J Shao J Luo S Nie S. Polymorphisms in PPARD, PPARG, and APM1 associated with four types of Traditional Chinese Medicine constitutions. J Genet Genomics. (2010) 37:371–9. 10.1016/S1673-8527(09)60055-2 20621019

[32] Wang Q. A series of studies on phlegm-dampness constitution and the application in prevention and control of chronic metabolic disease (in Chinese). Tianjin J Tradit Chin Med. (2020) 37:4–8. 10.11656/j.issn.1672-1519.2020.01.02

[33] Glazier RH. Association versus causation. Can Med Assoc J. (2018) 190:E863. 10.1503/cmaj.69466 30012804 PMC6050115

