# Supplementary material for: Etiology Exploration of Non-alcoholic Fatty Liver Disease From Traditional Chinese Medicine Constitution Perspective: A Cross-Sectional Study
Source: Front Public Health. 2021 May 12;9:635818. doi: 10.3389/fpubh.2021.635818 (PMC8149586; doi:10.3389/fpubh.2021.635818)
Supplement: Supplementary file 2 [file Table_2.DOCX]

**Chinese Medicine Constitution Questionnaire Designed by Wang Qi**

This questionnaire aims to survey your constitution and to subsequently provide a reference for future health management and clinical diagnosis. Please read every question carefully and choose the most suitable item based on your actual situation or feeling in the past year. If you are unsure of the answer to a specific question, choose the answer that is most similar to your actual situation. Make sure that you answer all the questions based on your situation **in the past year**, (excluding the effect of drugs) and give only one answer to each question.

Explanation: 【No】Never happened in the past year. 【Slightly】Occasionally happened in the past year. 【Sometimes】Sometimes it happened, but no regular pattern. 【Often】It happened most of the time in the past year. 【All the time】It happened all the time in the past year.

| Experience/condition in the past year | No | Slightly | Sometimes | Often | All the time |
| --- | --- | --- | --- | --- | --- |
| 1. Were you energetic? | 5 | 4 | 3 | 2 | 1 |
| 1. Did you get tired easily? | 5 | 4 | 3 | 2 | 1 |
| 1. Did you suffer from shortness of breath? | 5 | 4 | 3 | 2 | 1 |
| 1. Did you get palpitations? | 5 | 4 | 3 | 2 | 1 |
| 1. Did you get dizziness easily or become giddy when standing up? | 5 | 4 | 3 | 2 | 1 |
| 1. Did you prefer quietness and do not like to talk? | 5 | 4 | 3 | 2 | 1 |
| 1. Did you feel feeble when talking? | 5 | 4 | 3 | 2 | 1 |
| 1. Did you feel gloomy and depressed? | 5 | 4 | 3 | 2 | 1 |
| 1. Did you get anxious and worried easily? | 5 | 4 | 3 | 2 | 1 |
| 1. Did you feel sensitive, vulnerable or emotionally upset? | 5 | 4 | 3 | 2 | 1 |
| 1. Were you easily scared or frightened? | 5 | 4 | 3 | 2 | 1 |
| 1. Did you experience distention in the underarm or breast? | 5 | 4 | 3 | 2 | 1 |
| 1. Did you feel chest or stomach stuffiness? | 5 | 4 | 3 | 2 | 1 |
| 1. Did you sigh for no reason? | 5 | 4 | 3 | 2 | 1 |
| 1. Did your body feel heavy or lethargic? | 5 | 4 | 3 | 2 | 1 |
| 1. Did the palms of your hands or soles of your feet feel hot? | 5 | 4 | 3 | 2 | 1 |
| 1. Did your hands or feet feel cold or clammy? | 5 | 4 | 3 | 2 | 1 |
| 1. Did you feel cold easily in your abdomen, back, lower back or knees? | 5 | 4 | 3 | 2 | 1 |
| 1. Were you sensitive to cold and tend to wear more clothes than others? | 5 | 4 | 3 | 2 | 1 |
| 1. Did your body and face feel hot? | 5 | 4 | 3 | 2 | 1 |
| 1. Did you feel more vulnerable to the cold than others (winter coldness, air conditioners, fans, etc.)? | 5 | 4 | 3 | 2 | 1 |
| 1. Did you catch colds more easily than others? | 5 | 4 | 3 | 2 | 1 |
| 1. Did you sneeze even when you did not have a cold? | 5 | 4 | 3 | 2 | 1 |
| 1. Did you have runny or stuffy nose even when you did not have a cold? | 5 | 4 | 3 | 2 | 1 |
| 1. Did you cough due to seasonal change, temperature change, or unpleasant odor? | 5 | 4 | 3 | 2 | 1 |
| 1. Did you sweat easily when you had a slightly increased physical activity? | 5 | 4 | 3 | 2 | 1 |
| 1. Did you forget things easily? | 5 | 4 | 3 | 2 | 1 |
| 1. Did you have an excessively oily forehead and/or T-zone? | 5 | 4 | 3 | 2 | 1 |
| 1. Were your lips redder than others? | 5 | 4 | 3 | 2 | 1 |
| 1. Did you have allergies? (E.g. medicine, food, odors, pollen, pet dander, or during seasonal or weather change etc.?) | 5 | 4 | 3 | 2 | 1 |
| 1. Did your skin get hives/urticaria easily? | 5 | 4 | 3 | 2 | 1 |
| 1. Did your skin have purpura (purple spots, ecchymosis) due to allergies? | 5 | 4 | 3 | 2 | 1 |
| 1. Did black or purple bruises appear on your skin for no reason? | 5 | 4 | 3 | 2 | 1 |
| 1. Did your skin turn red and show traces when you scratched it? | 5 | 4 | 3 | 2 | 1 |
| 1. Did your skin or lips feel dry? | 5 | 4 | 3 | 2 | 1 |
| 1. Did you have visible capillary/thread veins on your cheeks? | 5 | 4 | 3 | 2 | 1 |
| 1. Did you feel pain somewhere in your body? | 5 | 4 | 3 | 2 | 1 |
| 1. Did you get hot flashes? | 5 | 4 | 3 | 2 | 1 |
| 1. Did your nose or your face feel greasy, oily, or shiny? | 5 | 4 | 3 | 2 | 1 |
| 1. Did you have a dark face or get brown spots easily? | 5 | 4 | 3 | 2 | 1 |
| 1. Did you get acne or sores easily? | 5 | 4 | 3 | 2 | 1 |
| 1. Did you have upper eyelid swelling? | 5 | 4 | 3 | 2 | 1 |
| 1. Did you get dark circles under the eyes easily? | 5 | 4 | 3 | 2 | 1 |
| 1. Did your eyes feel dry and use eye drops? | 5 | 4 | 3 | 2 | 1 |
| 1. Did your lips darker, more blue or purple then usual? | 5 | 4 | 3 | 2 | 1 |
| 1. Did you often feel parched and need to drink water? | 5 | 4 | 3 | 2 | 1 |
| 1. Did your throat feel strange (i.e., Like something was stuck or there was a lump in your throat)? | 5 | 4 | 3 | 2 | 1 |
| 1. Did you have bitterness or a strange taste in your mouth? | 5 | 4 | 3 | 2 | 1 |
| 1. Did your mouth feel sticky? | 5 | 4 | 3 | 2 | 1 |
| 1. Was your stomach/belly flabby? | 5 | 4 | 3 | 2 | 1 |
| 1. Did you have lots of phlegm, especially in your throat? | 5 | 4 | 3 | 2 | 1 |
| 1. Did you feel uncomfortable when you drank or ate something cold, or did you avoid drinking or eating something cold? | 5 | 4 | 3 | 2 | 1 |
| 1. Could you adapt yourself to external natural or social environment change? | 5 | 4 | 3 | 2 | 1 |
| 1. Did you suffer from insomnia? | 5 | 4 | 3 | 2 | 1 |
| 1. Did you easily get diarrhea when exposed to a cold or ate (or drank) something cold? | 5 | 4 | 3 | 2 | 1 |
| 1. Did you pass sticky stools and/or feel that your bowel movement is incomplete? | 5 | 4 | 3 | 2 | 1 |
| 1. Did you get constipated easily or have dry stools? | 5 | 4 | 3 | 2 | 1 |
| 1. Did your tongue have a thick coating? | 5 | 4 | 3 | 2 | 1 |
| 1. Did your urethral canal feel hot when you urinated, or did your urine have a dark color? | 5 | 4 | 3 | 2 | 1 |
| 1. Was your scrotum always wet (only for male interviewees)? Was your vaginal discharge yellowish (only for female interviewees)? | 5 | 4 | 3 | 2 | 1 |

Original scores: Sum up each item’s score

Converted scores: [(original scores - items)/(items*4)]* 100

Determination:

| Constitution | Condition | Result |
| --- | --- | --- |
| Balanced constitution | Converted scores>60 | Yes |
|  | The rest constitutions’ converted scores all<30 |  |
|  | Converted scores>60 | Basically yes |
|  | The rest constitutions’ converted scores all<40 |  |
|  | Not stratified the conditions above | No |
| Unbalanced constitution | Converted scores>40 | Yes |
|  | Converted scores39~30 | Tend to |
|  | Converted scores<30 | No |

Balanced constitution

Original scores = item 2 + item 7 + item 21 + item 27 + item 8 + item 1 + item 53 + item 54

Converted scores = [((item 2 + item 7 + item 21+ item 27 + item 8 + item 1 + item 53 + item 54)-8)/8*4]*100

Qi-deficiency constitution

Original scores = item 2 + item 3 + item 4 + item 5 + item 22 + item 6 + item 7+ item 26

Converted scores = [((item 2 + item 3 + item 4+ item 5 + item 22+ item 6+ item 7+ item 26)-8)/8*4]*100

Yang-deficiency constitution

Original scores = item 17 + item 18 + item 19 + item 21 + item 22 + item 52 + item 55

Converted scores = [(( item 17 + item 18 + item 19 + item 21 + item 22 + item 52 + item 55)-7)/7*4]*100

Yin-deficiency constitution

Original scores = item 16 + item 20 + item 35 + item 29 + item 57 + item 38 + item 44 + item 46 Converted scores = [(( item 16 + item 20 + item 35 + item 29 + item 57 + item 38 + item 44 + item 46)-8)/8*4]*100

Phlegm-dampness constitution

Original scores = item 13 + item 15 + item 50 + item 28 + item 42 + item 49 + item 51 + item 58

Converted scores = [(( item 13 + item 15 + item 50 + item 28 + item 42 + item 49 + item 51 + item 58)-8)/8*4]*100

Dampness-heat constitution

Original scores = item 39 + item 41 + item 48 + item 56 + item 59 + item 60

Converted scores = [(( item 39 + item 41 + item 48 + item 56 + item 59 + item 60)-6)/6*4]*100

Blood-stasis constitution

Original scores = item 33 + item 36 + item 37 + item 40 + item 43 + item 27 + item45

Converted scores =[(( item 33 + item 36 + item 37 + item 40 + item 43 + item 27 + item 45)-7)/7*4]*100

Qi-depression constitution

Original scores = item 8 + item 9 + item 10 + item 11 + item 12 + item 14 + item 47

Converted scores = [(( item 8 + item 9 + item 10 + item 11 + item 12 + item 14 + item 47)-7)/7*4]*100

Special diathesis constitution

Original scores = item 23 + item 24 + item 25 + item 30 + item 31 + item 32 + item 34

Converted scores =[(( item 23 + item 24 + item 25 + item 30 + item 31 + item 32 + item 34)-7)/7*4]*10
